# Virus Infection, Genetic Mutations, and Prion Infection in Prion Protein Conversion

**DOI:** 10.3390/ijms222212439

**Published:** 2021-11-18

**Authors:** Hideyuki Hara, Suehiro Sakaguchi

**Affiliations:** Division of Molecular Neurobiology, The Institute for Enzyme Research (KOSOKEN), Tokushima University, 3-18-15 Kuramoto, Tokushima 770-8503, Japan; hara@tokushima-u.ac.jp

**Keywords:** prion, prion protein, prion disease, neurodegenerative disease, virus infection, conformational conversion, influenza virus, protein polymerization

## Abstract

Conformational conversion of the cellular isoform of prion protein, PrP^C^, into the abnormally folded, amyloidogenic isoform, PrP^Sc^, is an underlying pathogenic mechanism in prion diseases. The diseases manifest as sporadic, hereditary, and acquired disorders. Etiological mechanisms driving the conversion of PrP^C^ into PrP^Sc^ are unknown in sporadic prion diseases, while prion infection and specific mutations in the PrP gene are known to cause the conversion of PrP^C^ into PrP^Sc^ in acquired and hereditary prion diseases, respectively. We recently reported that a neurotropic strain of influenza A virus (IAV) induced the conversion of PrP^C^ into PrP^Sc^ as well as formation of infectious prions in mouse neuroblastoma cells after infection, suggesting the causative role of the neuronal infection of IAV in sporadic prion diseases. Here, we discuss the conversion mechanism of PrP^C^ into PrP^Sc^ in different types of prion diseases, by presenting our findings of the IAV infection-induced conversion of PrP^C^ into PrP^Sc^ and by reviewing the so far reported transgenic animal models of hereditary prion diseases and the reverse genetic studies, which have revealed the structure-function relationship for PrP^C^ to convert into PrP^Sc^ after prion infection.

## 1. Introduction

Prion diseases, or transmissible spongiform encephalopathies, are a group of fatal neurodegenerative disorders in humans and animals, caused by accumulation of proteinaceous infectious particles, termed “prions”, in the brain [1,2,3]. Characteristic histopathological features, such as spongiform vacuolation, neuronal cell death, and gliosis, are observed in the affected brains [1,2]. Prion diseases manifest as sporadic, hereditary, or acquired disorders. Sporadic Creutzfeldt-Jakob disease (sCJD) is the most common prion disease in humans, accounting for 85–90% of total human cases [4,5,6]. The annual incidence of sCJD has been estimated to be 1–2 cases per a million people worldwide, although the reported incidence varies from country to country and is influenced by the method and extent of the study conducted [7]. 10–15% of human cases belong to hereditary prion diseases, such as familial CJD (fCJD), Gerstmann-Sträussler-Scheinker syndrome (GSS), and fatal familial insomnia (FFI) [8,9]. They are causatively linked to specific mutations in the gene (*Prnp*) of prion protein (PrP) [8,9]. The remaining cases, accounting for less than 1%, are those of acquired prion diseases, which include iatrogenic CJD (iCJD), variant CJD (vCJD), and kuru. iCJD is a disease caused by human-to-human transmission of prions via medical treatments or procedures [10]. Dura mater, cornea, and growth hormone from the patients, and depth electroencephalogram electrodes and neurosurgical instruments used for the patients have been considered to transmit the disease [11]. vCJD is believed to be transmitted from bovine spongiform encephalopathy (BSE) via oral consumption of BSE prion-contaminated food [10]. As of July 2021, 229 cases of vCJD have been identified worldwide since 1996, with most of them in the U.K. (http://www.cjd.ed.ac.uk/sites/default/files/worldfigs.pdf, accessed on 21 July 2021). Kuru is a disease spread via ritualistic cannibalism among Fore people in Papua New Guinea [11].

BSE is an acquired animal prion disease in cattle spread through contaminated meat-and-bone meals since the mid-1980s [12]. Transmissible mink encephalopathy in minks, chronic wasting disease (CWD) in deer, and feline spongiform encephalopathy in cats also acquired prion diseases [13,14]. Scrapie is a sporadic animal prion disease in sheep [13]. Unlike the case of BSE, epidemiological studies suggest unlikelihood of the zoonotic transmissions of scrapie or CWD to humans [15,16,17,18,19].

Prions are widely believed to consist of the abnormally folded, amyloidogenic isoform of PrP, designated PrP^Sc^, which is produced from the cellular isoform of PrP, PrP^C^, through its conformational conversion [1,2,3]. We and others have shown that mice devoid of PrP^C^ (*Prnp*^0/0^) are resistant to prion infections, neither developing disease nor propagating prions in the brains after intracerebral inoculation with the prions [20,21,22,23], confirming that the conversion of PrP^C^ into PrP^Sc^ eventually leading to accumulation of PrP^Sc^ in the brain is a key pathogenic event in prion diseases. In acquired prion diseases, prion infections drive the conversion of PrP^C^ into PrP^Sc^. Mutated PrPs in hereditary prion diseases may undergo conformational changes to form PrP^Sc^ [8,9]. We have recently reported that neurotrophic influenza A virus (IAV) caused the conversion of PrP^C^ into PrP^Sc^ in cultured neuronal cells after infection [24], raising the intriguing possibility that IAV infection in neurons might be a cause of or be associated with sporadic prion diseases.

Here, we present our current findings of the IAV infection-induced conversion of PrP^C^ into PrP^Sc^. We also review the so far reported transgenic (Tg) mouse models of hereditary prion diseases, where mutated PrPs are spontaneously converted into PrP^Sc^s, and the reverse genetic studies using *Prnp*^0/0^ mice, which have revealed the important regions for PrP^C^ to convert into PrP^Sc^ after prion infection.

## 2. PrP Conversion

### 2.1. PrP^C^ and PrP^Sc^

PrP^C^ is a host-encoded glycoprotein tethered to the plasma membrane via a glycosylphosphatidylinositol (GPI) anchor moiety and expressed most abundantly in the brain, particularly by neurons, and to lesser extents in other non-neuronal tissues, including heart, lung, pancreas, liver, spleen, testis, and kidney [25,26,27]. Many physiological functions have been suggested for PrP^C^, including oxidative stress mitigation, neuroprotection, and those associated with cell trafficking, cell adhesion, cell differentiation, cell signaling, and cell survival [28]. We have recently identified that PrP^C^ could also function as a protective molecule against IAV infection, mitigating oxidative stress and epithelial cell apoptosis in IAV-infected lungs, and a regulator molecule in macrophage M1/M2 polarization, stimulating the polarization to M2 anti-inflammatory macrophages [29,30].

PrP^C^ structurally consists of two domains; the flexible non-structural N-terminal domain including the so-called octapeptide repeat (OR) region in the middle, and the globular C-terminal domain with 2 short β-sheets and 3 α-helices [26,27]. Mouse PrP^C^ is translated as a precursor protein comprised of 254 amino acids, with the 22 amino acids of an endoplasmic reticulum (ER) migration signal sequence at the N-terminus and the 23 amino acids of a GPI anchor addition signal sequence at the C-terminus [26,27]. Both signal sequences are removed in the ER as the nascently translated PrP enters the ER [26,27]. The C-terminal domain further undergoes posttranslational modifications, such as disulfide bond formation between cysteine residues at positions 178 and 213 and N-type sugar chain addition at asparagine residues of positions 180 and 196 [26,27].

PrP^Sc^ is specifically detectable in the tissues or cells infected with prions, including the central nervous system, particularly neurons, and follicular dendric cells of the lymphoreticular system [31]. In contrast to PrP^C^ being detergent-soluble and sensitive to protease degradation, PrP^Sc^ is highly detergent-insoluble, easily assembling to form amyloid fibrils, termed prion rods, with a diameter of 10–20 nm and a length of 100–200 nm, and relatively resistant to protease degradation [32]. These biochemical differences of both molecules arise from their structural differences. PrP^C^ is rich in α-helix contents while PrP^Sc^ abounds with β-sheet contents [33].

### 2.2. In Vitro PrP Conversion

Kocisko et al. succeeded for the first time in producing PrP^Sc^-like proteinase K (PK)-resistant PrP from PrP^C^ in vitro, by incubating PrP^C^ from prion-uninfected cultured cells with PrP^Sc^ partially purified from 263K prion-infected hamster brains in denaturing-renaturing conditions [34]. The protein misfolding cyclic amplification (PMCA) technique was then developed to amplify PrP^Sc^-like PrP in vitro by serially incubating PrP^C^ with the diluted PMCA products under sonication, eventually enriching PrP^Sc^-like PrP converted from PrP^C^ and contrarily reducing original PrP^Sc^ added in the reactions to a negligible level [35]. Subsequently, PrP^Sc^-like PrP generated by PMCA was shown to correlate to prion infectivity, strongly suggesting that PrP^Sc^-like PrP is generated by PMCA is infectious [36]. Prion infectivity was also successfully generated from bacterial recombinant PrP without incubating with PrP^Sc^. The β sheet-rich amyloid fibrils consisting of the N-terminally truncated recombinant mouse PrP were shown to be infectious [37]. PMCA with bacterial recombinant full-length mouse PrP together with the synthetic anionic phospholipid and RNA molecules was also shown to generate PrP^Sc^-like PrP as well as having prion infectivity [38]. Dextran sulfate, mRNA molecules, and plasmid DNA were also reported to function as co-factors to produce infectious prions from bank vole PrP in PMCA, although bank vole PrP was convertible into infectious prions in PMCA without these cofactors [39]. These results confirm that PrP^Sc^ is an essential component for prions to be infectious, strongly supporting that prions consist of PrP^Sc^ alone. Consistent with this, prion infectivity has been shown to be sensitive to treatments with protein-denaturing agents, but resistant to nucleic acid-degrading treatments with nucleases or UV radiation [40]. No specific nucleic acids have been isolated in the highly purified prion fractions [41].

### 2.3. Seeded-Polymerization Mechanism in PrP Conversion

**Figure 1:**
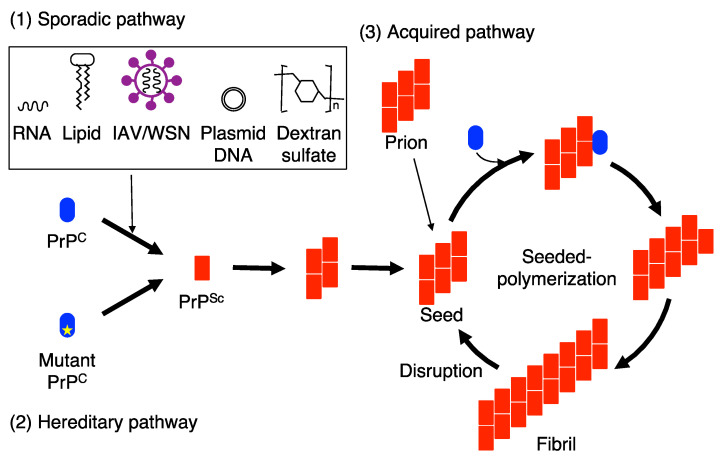
Seeded protein polymerization model for the conversion of PrP^C^ into PrP^Sc^ in sporadic, hereditary, and acquired prion diseases. (1) IAV/WSN infection and interaction with RNA, lipid molecules plasmid DNA and dextran sulfate drives PrP^C^ to undergo conformational changes to form PrP^Sc^ in sporadic prion diseases. (2) In hereditary prion diseases, mutated PrPs are prone to convert into PrP^Sc^. The nascently generated PrP^Sc^ molecules in sporadic and hereditary prion diseases assemble to form small oligomeric PrP^Sc^ aggregates and then further polymerize to form PrP^Sc^ seeds, which are able to recruit and force PrP^C^ to convert into PrP^Sc^ on them. (3) In acquired prion diseases, exogenously invading prions function as PrP^Sc^ seeds for the conversion of PrP^C^ into PrP^Sc^. As a result, the PrP^Sc^ oligomers are elongated to form fibrils in sporadic, hereditary, and acquired prion diseases. The fibrils are then disrupted into prions, or small PrP^Sc^ seeds, which then enter the conversion cycle of PrP^C^ into PrP^Sc^.

It has been suggested that a seeded-protein polymerization mechanism is an underlying mechanism for PrP^Sc^ propagation, or prion propagation [34,42]. Prion-infected brain fractions containing the aggregates of 14–28 PrP^Sc^ molecules were shown to be highly infectious compared to those containing large PrP^Sc^ fibrils or the aggregates of less than 5 PrP^Sc^ molecules [43], suggesting that prions are the multimeric aggregates of PrP^Sc^. In the seeded-protein polymerization mechanism, the aggregates of PrP^Sc^ are considered to function as a seed or a scaffold for PrP^C^ to undergo conformational conversion into PrP^Sc^ (Figure 1). PrP^C^ is recruited onto the PrP^Sc^ seed and forced to convert into PrP^Sc^ on it through structural transition from α-helix to β-sheet [33], resulting in elongation of the PrP^Sc^ seeds, eventually leading to formation of the PrP^Sc^ fibrils (Figure 1). Prions, or PrP^Sc^ seeds, are postulated to then propagate by cleavage of the PrP^Sc^ fibrils into new multiple PrP^Sc^ seeds by unknown cellular mechanism (Figure 1) [34,35].

Structural models have been proposed for PrP^Sc^ fibrils. In a 4-rung β-solenoid model, a PrP^Sc^ molecule adopts a solenoid structure with 4 rungs, each rung consisting of 3 β-sheets and perpendicularly stacking with each other to form a PrP^Sc^ protofilament [44,45,46]. Two PrP^Sc^ protofilaments are then intertwined, forming a PrP^Sc^ fibril [44,45,46]. The PrP^Sc^ protofilaments might function as seeds, recruiting PrP^C^ molecules to their upper and lower ß-solenoid rungs and inducing the formation of ß-solenoid rungs through the structural transition of α-helices to β-sheet, eventually converting the PrP^C^ into PrP^Sc^. In a parallel in-register intermolecular ß-sheet model, a PrP^Sc^ fibril is formed by stacking PrP^Sc^ molecules, each PrP^Sc^ comprising the entire cross-section of the fibril with many hairpins, through the intermolecular β-sheet interactions between them [47,48]. The fibrils function as seeds for PrP^C^ to convert into PrP^Sc^.

## 3. Virus Infections in PrP Conversion

### 3.1. Neurotropic IAV Infection in PrP Conversion

We have recently reported that infection of mouse neuroblastoma N2a cells overexpressing the exogenously transduced mouse PrP^C^, termed N2aC24 cells, with a neurotropic strain of influenza A/WSN/33 (H1N1) virus (hereafter referred to as IAV/WSN) induced spontaneous conformational changes in PrP^C^ to form PrP^Sc^-like PK-resistant PrP [24]. These results indicate that, like prion infection, IAV/WSN infection could induce the conformational conversion of PrP^C^ into PrP^Sc^. We also detected PrP^Sc^-like PrP in parent N2a cells, which express endogenous mouse PrP^C^ alone, after infection with IAV/WSN [24], indicating that overexpression of PrP^C^ is not required for the IAV/WSN-induced conversion into PrP^Sc^. We further showed that the anti-IAV agent oseltamivir and anti-IAV mouse antisera blocked IAV/WSN infection of N2aC24 cells and prevented formation of PrP^Sc^-like PrP in the cells [24], not only ruling out the possibility of the contamination of laboratory prions in the cell culture, but also confirming that IAV/WSN infection is essential for the conversion of PrP^C^ into PrP^Sc^-like PrP in the cells. We finally demonstrated that wild-type (WT) mice intracerebrally inoculated with cell lysates from PrP^Sc^-like PrP-producing N2aC24 cells developed prion disease, with abundant accumulation of PrP^Sc^ and spongiosis in their brains [24], indicating that, like authentic PrP^Sc^ produced by prion infection, PrP^Sc^-like PrP produced in IAV/WSN-infected N2aC24 cells could form infectious prions. Taken together, these results suggest that IAV infection in neurons might be a cause of or be associated with sporadic prion diseases, including sCJD.

PrP^Sc^-like PrP (hereafter simply referred to as PrP^Sc^) was constitutively produced in IAV/WSN-infected N2aC24 cells even after IAV/WSN infection was cleared during long culture [24], indicating that persistent IAV/WSN infection is not necessary for the constitutive conversion PrP^C^ into PrP^Sc^ in the cells. It is thus possible that IAV/WSN infection could induce the conversion of PrP^C^ into PrP^Sc^ in a hit-and-run manner, and the nascent PrP^Sc^ could polymerize to form the PrP^Sc^ seeds, enabling the constitutive conversion of PrP^C^ into PrP^Sc^ in the seeded-polymerization mechanism. IAVs are negative-stranded, segmented, enveloped RNA viruses [49]. It was reported that RNA molecules were able to convert human recombinant PrP into PK-resistant PrP in vitro [50]. Moreover, RNA molecules were shown to function as a cofactor, together with the synthetic anionic phospholipid, for the conversion of recombinant PrP into PrP^Sc^ in PMCA [38]. These results indicate that RNA molecules play an important role in the conversion of PrP^C^ into PrP^Sc^. IAV/WSN-derived RNA molecules might play the same role in the conversion of PrP^C^ into PrP^Sc^ in N2aC24 cells. However, it has been reported that RNA molecules prepared from mammalian tissues but not from invertebrate species including bacteria, yeast, worms, and flies converted PrP^C^ into PrP^Sc^ in PMCA [51]. For elucidation of the role of IAV/WSN-derived RNA molecules in the conversion of PrP^C^ into PrP^Sc^, it would be helpful to investigate if IAV/WSN-derived RNA molecules have an ability to convert PrP^C^ into PrP^Sc^ in PMCA.

### 3.2. Other Virus Infections in Prion Infection

It was shown that mice co-infected with scrapie prions and mouse adenovirus accelerated prion disease compared to those infected with scrapie prions alone, suggesting that mouse adenovirus infection could enhance prion pathogenesis in mice [52]. Infection with the Cork strain of caprine arthritis encephalitis virus, a small-ruminant lentivirus, was also shown to increase PrP^Sc^ in scrapie prion-infected primary sheep microglia at levels 2-times higher than in control cells [53]. However, no data were available as to whether these virus infections could cause the de novo conversion of PrP^C^ into PrP^Sc^ [53]. Mouse A9 fibroblasts infected with murine minute virus, a murine parvovirus, were reported to bind to exogenously added PrP^Sc^ molecules more abundantly and internalize them to lysosomal compartments, which are postulated to be one of the cellular sites for the conversion of PrP^C^ into PrP^Sc^, more efficiently than control cells [54]. Molony murine leukemia virus infection into 22L scrapie prion-infected NIH3T3 cells was reported to increase the release of PrP^Sc^ in culture medium by facilitating the incorporation of PrP^Sc^ into virus particles or exosomes [55]. It is thus possible that the increased levels of PrP^Sc^ in the prion-infected cells after infection with mouse adenovirus or caprine arthritis encephalitis virus might be due to the increased accessibility of PrP^Sc^ to PrP^C^ through the enhanced binding of PrP^Sc^ molecules on the cells, their stimulated internalization to lysosomal compartments, and the increased release of PrP^Sc^ from prion-infected cells.

## 4. Tg Mouse Models of Hereditary Prion Diseases

### 4.1. Genetic Mutations in PrP Conversion

**Figure 2:**
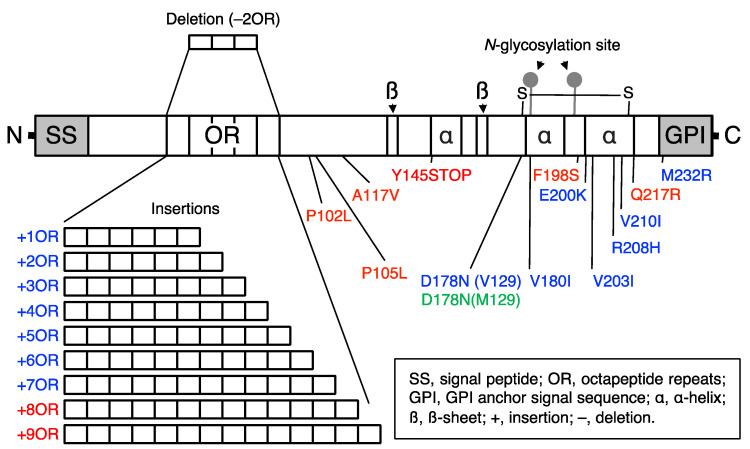
Schematic illustration of hereditary prion disease-associated mutations in PrP. GSS, fCJD, and FFI mutations are indicated in red, blue, and green, respectively.

Many point mutations and insertions or a deletion of the OR sequence have been identified in hereditary human prion diseases, including GSS, fCJD, and FFI (Figure 2) [8,9]. To understand the pathogenic mechanism of hereditary prion diseases, many lines of Tg mice expressing the disease-associated mutant PrPs have been reported, uncovering biologically and structurally different forms of PrP^Sc^, or neurotoxic and infectious PrP^Sc^s [56]. Some PrP mutants were shown to convert into neurotoxic forms but not to infectious forms while others converted to neurotoxic and infectious forms [56]. It is thus possible that PrP^C^ might undergo conformational changes to convert into PrP^Sc^, then polymerizing to form neurotoxic PrP^Sc^ oligomers and to neurotoxic and infectious PrP^Sc^ oligomers, or PrP^Sc^ fibrils.

### 4.2. Tg Mouse Models of GSS

The first reported Tg mouse model of hereditary prion diseases is Tg(PrP-P101L) mice, which express mouse PrP-P101L, the analogous mutation to human PrP-P102L in GSS (Table 1) [57]. They expressed the mutant PrP at levels at least 3 times higher than PrP^C^ in WT mice and spontaneously developed prion disease-like disease at 150–600 days after birth, with the disease-characteristic pathologies of amyloid plaques, spongiform degeneration, and gliosis in their brains [57]. However, in contrast to authentic PrP^Sc^, which is highly resistant to PK, PrP^Sc^-P101L accumulated in the brains of ill Tg(PrP-P101L) mice was easily digested by a high dose of PK [57]. In addition, intracerebral inoculation with brain homogenates from ill Tg(PrP-P101L) mice transmitted the disease to 40% of Tg(PrP-P101L) mice, which express low levels of the mutant protein, and 10% of hamsters, but not to WT mice [58]. These results suggest that PrP-P101L could convert into neurotoxic and infectious PrP^Sc^-P101L. However, the infectivity of PrP^Sc^-P101L was dependent on recipient animals. Tg(PrP-A116V) mice, which express mouse PrP-A116V (the human homologue of PrP-A117V in GSS) at levels 4–6 times higher than PrP^C^ in WT mice, also spontaneously developed neurological disease around 150 days after birth, with spongiosis and PrP amyloid plaques in their brains (Table 1) [59]. PrP^Sc^-A117V in the brains of ill Tg(PrP-A116V) mice was partially detergent-insoluble and weakly resistant to PK [59]. No data are available as to whether PrP^Sc^-A117V is infectious [59]. Tg(PG14)/*Prnp*^0/0^ mice are another GSS mouse model expressing a mutant PrP with 9 extra OR sequences in the OR region on the genetic background of *Prnp*^0/0^ (Table 1) [60,61]. These mice developed spontaneous cerebellar neurodegeneration, with granule cell death and very slight but substantial accumulation of PrP^Sc^-PG14 in their brains [60,61]. PrP^Sc^-PG14 showed weak resistance to PK and had no prion infectivity in animals [62]. These results indicate that PrP-PG14 could convert into neurotoxic PrP^Sc^-PG14 but not into infectious PrP^Sc^-PG14.

### 4.3. Tg Mouse Models of fCJD

Tg(Mo/Hu-PrP-E199K) mice, which express mouse-human chimeric PrP, or Mo/Hu-PrP, with the E199K mutation (the human homologue of E200K in fCJD) 2 times higher than PrP^C^ in WT mice, were reported to develop neurological disease around 200 days of age, with hind-limb paralysis and kyphosis (Table 1) [63]. Mo/Hu-PrP^Sc^-E199K in the brains of sick Tg(Mo/Hu-PrP-E199K) mice showed atypical PK-resistant bands on Western blotting [63]. Inoculation of brain homogenates from sick Tg(Mo/Hu-PrP-E199K) mice transmitted the disease into non-Tg mice with highly variable efficiency [63]. These results indicate that Mo/Hu-PrP-E199K could convert into neurotoxic and infectious Mo/Hu-PrP^Sc^-E199K. However, interestingly, Tg mice overexpressing human PrP with the E200K mutation in the brains at levels 2 to 3 times higher than PrP^C^ in human brains developed no neurological diseases (Table 1) [64]. In contrast, knock-in mice expressing mouse PrP-E199K with the 3F4 epitope, which derives from human PrP, exhibited abnormal behaviors, such as reduced performance on the rotarod test and decreased burrowing behavior, with spongiosis and punctuate deposition of PrP^Sc^-E199K(3F4) in their brains (Table 1) [65]. PrP^Sc^-E199K(3F4) was weakly resistant to PK [65]. Inoculation of brain homogenates from sick Tg[PrP-E199K(3F4)] mice transmitted the disease in Tg mice overexpressing Mo-PrP as well as in non-Tg mice [65]. It is thus possible that the human PrP-derived 3F4 sequence might affect mouse PrP to spontaneously convert into neurotoxic and infectious PrP^Sc^.

Tg mice overexpressing Mo-PrP with the D177N mutation (the human homologue of D178N in fCJD) along with the polymorphic amino acid substitution of M128V (the human homologue of M129V) 2 times higher than PrP^C^ in WT mice were also reported to develop neurological disease around 150 days of age, with ataxia, kyphosis, and foot clasping (Table 1) [66]. PrP^Sc^-D177N(V128) was detergent-insoluble and mildly resistant to PK [66]. Inoculation of brain homogenates from sick Tg[PrP-D177N(V128)] mice failed to transmit the disease to non-Tg mice as well as Tg mice overexpressing mouse PrP and Tg mice expressing lower levels of PrP-D177N(V128) [67]. These results suggest that PrP-D177N(V128) could convert into neurotoxic PrP^Sc^-D177N(V128) but not to infectious PrP^Sc^-D177N(V128).

### 4.4. Tg Mouse Models of FFI

Tg(FFI)/*Prnp*^0/0^ mice, which express mouse PrP-D177N(M128) corresponding to the human homologue of PrP-D178N(M129) in FFI in their brains 2 times higher than PrP^C^ in WT mice, were reported to develop fatal neurological disease with motor and cognitive deficits (Table 1) [67]. Circadian rhythms of sleep and motor activity were also disturbed in Tg(FFI)/*Prnp*^0/0^ mice, as observed in FFI patients [67]. Sick Tg(FFI)/*Prnp*^0/0^ mice accumulated weakly PK-resistant PrP^Sc^-D177N(M128) in their brains [67]. Inoculation of brain homogenates from sick Tg(FFI)/*Prnp*^0/0^ mice failed to transmit the disease to WT mice and Tg mice overexpressing mouse PrP [67]. These results suggest that PrP-D177N(M128) could convert into neurotoxic PrP^Sc^-D177N(M128) but not to infectious PrP^Sc^-D177N(M128). However, knock-in mice expressing PrP-D178N(M129) with the 3F4 epitope sequence were shown to develop neurological disease with neuronal loss and gliosis in the thalamus and transmit the disease to Tg mice overexpressing mouse PrP after inoculation with their brain homogenates (Table 1) [68]. It is thus suggested that the 3F4 sequence could force PrP-D178N(M129) to undergo conformational changes to form neurotoxic PrP^Sc^-D177N(M128) and then to neurotoxic and infectious PrP^Sc^-D177N(M128).

## 5. Reverse Genetic Studies for Acquired Prion Diseases

### 5.1. The N-Terminal Polybasic Residues of PrP^C^ in Prion Infection

Successful restoration of the susceptibility to prion infection in *Prnp*^0/0^ mice by transgenic expression of full-length mouse PrP^C^ has enabled reverse genetic studies to identify the regions or amino acids in PrP^C^ important for the conversion into PrP^Sc^ after prion infection [69]. Tg(PrP∆23–31)/*Prnp*^0/0^ mice, which express PrP with a deletion of the so-called N-terminal polybasic region consisting of residues 23–31 on the background of *Prnp*^0/0^, were shown to develop disease after longer incubation times with a slower accumulation of PrP^Sc^∆23–31 in their brains after infection with RML scrapie prions, indicating that the polybasic region is important for PrP^C^ to convert into PrP^Sc^ after prion infection (Table 2) [70]. We generated Tg(PrP3K3A)/*Prnp*^0/0^ mice, which express mouse PrP^C^ with lysine residues at positions 23, 24 and 27 mutated to alanine residues in the polybasic region, and intracerebrally inoculated them with RML and 22L scrapie prions (Table 2) [71]. They were highly resistant to the prions, developing disease with longer incubation times [71], suggesting that lysine residues at 23, 24, and 27 are important for the polybasic region to support prion infection. Recombinant PrP∆23-31 was weakly interacted with PrP^Sc^ compared to full-length PrP [70]. The polybasic region might thus be involved in the intermolecular interaction with PrP^C^ and PrP^Sc^ through the positively charged lysine residues, thereby supporting prion infection through driving the conversion of PrP^C^ into PrP^Sc^.

### 5.2. The Central Residues of PrP^C^ in Prion Infection

Tg(PrP∆32–80)/*Prnp*^0/0^ mice, which express mouse PrP lacking residues 32–80 including most of the OR region, developed disease without prolonged incubation times after infection with RML prions and accumulated PrP^Sc^∆32–80 in their brains (Table 2) [69]. However, Tg(PrP∆32–93)/*Prnp*^0/0^ mice expressing PrP with the deletion of residues 32–93 encompassing the entire OR region exhibited longer incubation times and reduced PrP^Sc^∆32–93 in their brains after infection with RML prions (Table 2) [72]. Furthermore, Tg(PrP∆32–106)/*Prnp*^0/0^ mice expressing a mutant PrP with a deletion extended to residue 106 neither developed disease nor accumulated PrP^Sc^∆32–106 in their brains even after intracerebral inoculation with RML prions (Table 2) [73]. These results suggest that the deletion of residues 91–106 not the OR region (residues 51–90) renders PrP∆32–106 unsusceptible to RML prions. Consistent with this, we demonstrated that, while Tg(PrP∆OR)/*Prnp*^0/0^ mice expressing PrP lacking the OR region alone were susceptible to RML and 22L prions (Table 2) [74], Tg(PrP∆91–106)/*Prnp*^0/0^ mice expressing PrP lacking residues 91–106 were resistant to RML and 22L prions, neither developing disease nor accumulating PrP^Sc^∆91–106 in their brains (Table 2) [75]. Using RML prion-infected N2a cells, ScN2a, it was shown that residues 100–104 are important for PrP^C^ to convert into PrP^Sc^ in the cells [76]. Residues 100–104 form a loop between the β-sheets in the PrP^Sc^ structural model [77,78,79]. It is thus possible that the loop region might play an important role in the conversion of PrP^C^ to PrP^Sc^ after infection with RML and 22L prions. Intriguingly, in contrast to the case for RML and 22L prions, Tg(PrP∆91–106)/*Prnp*^0/0^ mice were susceptible to BSE prions and Tg(PrP∆OR)/*Prnp*^0/0^ mice had reduced their susceptibility to BSE prions (Table 2) [74,75]. These results indicate that, in contrast to RML and 22L prions, BSE prions might require the OR region partially but not residues 91–106 to induce the conversion of PrP^C^ to PrP^Sc^, suggesting that the OR region and residues 91–106 of PrP^C^ are involved in prion infection in a prion strain-dependent manner.

## 6. Perspectives

The etiologies of sporadic prion diseases including sCJD remain unknown. Our findings that IAV/WSN infection induced the conversion of PrP^C^ into PrP^Sc^ and infectious prions in N2a cells has raised the possibility that IAV infection in neurons might be an etiological mechanism of sporadic prion diseases [24]. Elucidation of (1) the mechanism of how IAV/WSN infection could induce the conversion of PrP^C^ into PrP^Sc^, (2) whether or not IAV/WSN infection in neurons could induce prion disease in animals, and (3) whether or not IAV infection in humans could be epidemiologically linked to sCJD are crucial for further understanding of the causative role of IAV infection in sporadic prion diseases. Also, addressing (1) the mechanism of the conversion of the hereditary prion disease-associated mutated PrPs into PrP^Sc^, (2) the molecular nature of neurotoxic and infectious PrP^Sc^ molecules, and (3) the mechanism of how the N-terminal polybasic region and the central residues 91–106 are involved in the conversion of PrP^C^ into PrP^Sc^ is important not only for further understanding of the pathogenic mechanisms of prion diseases, but also for the development of effective treatment and prevention measures for prion diseases.

## Figures and Tables

**Table 1:** Hereditary prion disease-associated mutations in PrP conversion.

Hereditary Prion Diseases	Mutant PrPs	Conversion to Neurotoxic PrP^Sc^/Infectious PrP^Sc^	References
GSS	Mo-PrP-P101L	Yes/Yes	[57,58]
	Mo-PrP-A116V	Yes/N.D.	[59]
	Mo-PrP-PG14	Yes/No	[60,61,62]
fCJD	Mo/Hu-PrP-E199K	Yes/Yes	[63]
	Hu-PrP-E200K	No/No	[64]
	Mo-PrP-E199K(3F4)	Yes/Yes	[65]
	Mo-PrP-D177N(V128)	Yes/No	[66]
FFI	Mo-PrP-D177N(M128)	Yes/No	[67]
	Mo-PrP-D177N(M128)(3F4)	Yes/Yes	[68]

Mo, mouse; Hu, human; N.D., not determined.

**Table 2:** PrP deletional and point mutants in prion infections.

PrPs	Susceptibility to Prions	References
PrP∆23–31	Reduced to RML prions	[70]
PrP3K3A	Reduced to RML and 22L prions	[71]
PrP∆32–80	Not reduced to RML prions	[72]
PrP∆32–93	Reduced to RML prions	[73]
PrP∆32–106	Resistant to RML prions	[73]
PrP∆OR	Not reduced to RML and 22L prions Reduced to BSE prions	[74]
PrP∆91–106	Resistant to RML and 22L prions Reduced to BSE prions	[75]

## References

[1] Prusiner S.B. (1998). Prions. Proc. Natl. Acad. Sci. USA.

[2] DeArmond S.J., Prusiner S.B. (1995). Etiology and pathogenesis of prion diseases. Am. J. Pathol..

[3] Scheckel C., Aguzzi A. (2018). Prions, prionoids and protein misfolding disorders. Nat. Rev. Genet..

[4] Brandel J.P., Peckeu L., Haik S. (2013). The French surveillance network of Creutzfeldt-Jakob disease. Epidemiological data in France and worldwide. Transfus. Clin. Biol..

[5] Heinemann U., Krasnianski A., Meissner B., Varges D., Kallenberg K., Schulz-Schaeffer W.J., Steinhoff B.J., Grasbon-Frodl E.M., Kretzschmar H.A., Zerr I. (2007). Creutzfeldt-Jakob disease in Germany: A prospective 12-year surveillance. Brain.

[6] Maddox R.A., Person M.K., Blevins J.E., Abrams J.Y., Appleby B.S., Schonberger L.B., Belay E.D. (2020). Prion disease incidence in the United States: 2003–2015. Neurology.

[7] Uttley L., Carroll C., Wong R., Hilton D.A., Stevenson M. (2020). Creutzfeldt-Jakob disease: A systematic review of global incidence, prevalence, infectivity, and incubation. Lancet Infect. Dis..

[8] Webb T.E., Poulter M., Beck J., Uphill J., Adamson G., Campbell T., Linehan J., Powell C., Brandner S., Pal S. (2008). Phenotypic heterogeneity and genetic modification of P102L inherited prion disease in an international series. Brain.

[9] Cracco L., Appleby B.S., Gambetti P. (2018). Fatal familial insomnia and sporadic fatal insomnia. Handb. Clin. Neurol..

[10] Will R.G., Ironside J.W., Zeidler M., Cousens S.N., Estibeiro K., Alperovitch A., Poser S., Pocchiari M., Hofman A., Smith P.G. (1996). A new variant of Creutzfeldt-Jakob disease in the UK. Lancet.

[11] Will R.G. (2003). Acquired prion disease: Iatrogenic CJD, variant CJD, kuru. Br. Med. Bull..

[12] Wells G.A., Scott A.C., Johnson C.T., Gunning R.F., Hancock R.D., Jeffrey M., Dawson M., Bradley R. (1987). A novel progressive spongiform encephalopathy in cattle. Vet. Rec..

[13] Orge L., Lima C., Machado C., Tavares P., Mendonca P., Carvalho P., Silva J., Pinto M.L., Bastos E., Pereira J.C. (2021). Neuropathology of Animal Prion Diseases. Biomolecules.

[14] Williams E.S., Young S. (1982). Spongiform encephalopathy of Rocky Mountain elk. J. Wildl. Dis..

[15] Wilson R., Plinston C., Hunter N., Casalone C., Corona C., Tagliavini F., Suardi S., Ruggerone M., Moda F., Graziano S. (2012). Chronic wasting disease and atypical forms of bovine spongiform encephalopathy and scrapie are not transmissible to mice expressing wild-type levels of human prion protein. J. Gen. Virol..

[16] Belay E.D., Maddox R.A., Williams E.S., Miller M.W., Gambetti P., Schonberger L.B. (2004). Chronic wasting disease and potential transmission to humans. Emerg. Infect. Dis..

[17] Belay E.D., Gambetti P., Schonberger L.B., Parchi P., Lyon D.R., Capellari S., McQuiston J.H., Bradley K., Dowdle G., Crutcher J.M. (2001). Creutzfeldt-Jakob disease in unusually young patients who consumed venison. Arch. Neurol..

[18] Brown P., Cathala F., Raubertas R.F., Gajdusek D.C., Castaigne P. (1987). The epidemiology of Creutzfeldt-Jakob disease: Conclusion of a 15-year investigation in France and review of the world literature. Neurology.

[19] Van Duijn C.M., Delasnerie-Laupretre N., Masullo C., Zerr I., de Silva R., Wientjens D.P., Brandel J.P., Weber T., Bonavita V., Zeidler M. (1998). Case-control study of risk factors of Creutzfeldt-Jakob disease in Europe during 1993-95. European Union (EU) Collaborative Study Group of Creutzfeldt-Jakob disease (CJD). Lancet.

[20] Bueler H., Aguzzi A., Sailer A., Greiner R.A., Autenried P., Aguet M., Weissmann C. (1993). Mice devoid of PrP are resistant to scrapie. Cell.

[21] Prusiner S.B., Groth D., Serban A., Koehler R., Foster D., Torchia M., Burton D., Yang S.L., DeArmond S.J. (1993). Ablation of the prion protein (PrP) gene in mice prevents scrapie and facilitates production of anti-PrP antibodies. Proc. Natl. Acad. Sci. USA.

[22] Manson J.C., Clarke A.R., McBride P.A., McConnell I., Hope J. (1994). PrP gene dosage determines the timing but not the final intensity or distribution of lesions in scrapie pathology. Neurodegeneration.

[23] Sakaguchi S., Katamine S., Shigematsu K., Nakatani A., Moriuchi R., Nishida N., Kurokawa K., Nakaoke R., Sato H., Jishage K. (1995). Accumulation of proteinase K-resistant prion protein (PrP) is restricted by the expression level of normal PrP in mice inoculated with a mouse-adapted strain of the Creutzfeldt-Jakob disease agent. J. Virol..

[24] Hara H., Chida J., Uchiyama K., Pasiana A.D., Takahashi E., Kido H., Sakaguchi S. (2021). Neurotropic influenza A virus infection causes prion protein misfolding into infectious prions in neuroblastoma cells. Sci. Rep..

[25] Oesch B., Westaway D., Walchli M., McKinley M.P., Kent S.B., Aebersold R., Barry R.A., Tempst P., Teplow D.B., Hood L.E. (1985). A cellular gene encodes scrapie PrP 27-30 protein. Cell.

[26] Prusiner S.B. (1991). Molecular biology of prion diseases. Science.

[27] Hackl S., Becker C.F.W. (2019). Prion protein-Semisynthetic prion protein (PrP) variants with posttranslational modifications. J. Pept. Sci..

[28] Aguzzi A., Baumann F., Bremer J. (2008). The prion’s elusive reason for being. Annu. Rev. Neurosci..

[29] Chida J., Hara H., Yano M., Uchiyama K., Das N.R., Takahashi E., Miyata H., Tomioka Y., Ito T., Kido H. (2018). Prion protein protects mice from lethal infection with influenza A viruses. PLoS Pathog..

[30] Chida J., Hara H., Uchiyama K., Takahashi E., Miyata H., Kosako H., Tomioka Y., Ito T., Horiuchi H., Matsuda H. (2020). Prion protein signaling induces M2 macrophage polarization and protects from lethal influenza infection in mice. PLoS Pathog..

[31] Prusiner S.B. (1998). The prion diseases. Brain Pathol..

[32] Prusiner S.B., McKinley M.P., Bowman K.A., Bolton D.C., Bendheim P.E., Groth D.F., Glenner G.G. (1983). Scrapie prions aggregate to form amyloid-like birefringent rods. Cell.

[33] Pan K.M., Baldwin M., Nguyen J., Gasset M., Serban A., Groth D., Mehlhorn I., Huang Z., Fletterick R.J., Cohen F.E. (1993). Conversion of alpha-helices into beta-sheets features in the formation of the scrapie prion proteins. Proc. Natl. Acad. Sci. USA.

[34] Kocisko D.A., Come J.H., Priola S.A., Chesebro B., Raymond G.J., Lansbury P.T., Caughey B. (1994). Cell-free formation of protease-resistant prion protein. Nature.

[35] Saborio G.P., Permanne B., Soto C. (2001). Sensitive detection of pathological prion protein by cyclic amplification of protein misfolding. Nature.

[36] Castilla J., Saa P., Hetz C., Soto C. (2005). In vitro generation of infectious scrapie prions. Cell.

[37] Legname G., Baskakov I.V., Nguyen H.O., Riesner D., Cohen F.E., DeArmond S.J., Prusiner S.B. (2004). Synthetic mammalian prions. Science.

[38] Wang F., Wang X., Yuan C.G., Ma J. (2010). Generating a prion with bacterially expressed recombinant prion protein. Science.

[39] Fernandez-Borges N., Di Bari M.A., Erana H., Sanchez-Martin M., Pirisinu L., Parra B., Elezgarai S.R., Vanni I., Lopez-Moreno R., Vaccari G. (2018). Cofactors influence the biological properties of infectious recombinant prions. Acta Neuropathol..

[40] Prusiner S.B. (1982). Novel proteinaceous infectious particles cause scrapie. Science.

[41] Bolton D.C., McKinley M.P., Prusiner S.B. (1982). Identification of a protein that purifies with the scrapie prion. Science.

[42] Jarrett J.T., Lansbury P.T. (1993). Seeding “one-dimensional crystallization” of amyloid: A pathogenic mechanism in Alzheimer’s disease and scrapie?. Cell.

[43] Silveira J.R., Raymond G.J., Hughson A.G., Race R.E., Sim V.L., Hayes S.F., Caughey B. (2005). The most infectious prion protein particles. Nature.

[44] Wille H., Bian W., McDonald M., Kendall A., Colby D.W., Bloch L., Ollesch J., Borovinskiy A.L., Cohen F.E., Prusiner S.B. (2009). Natural and synthetic prion structure from X-ray fiber diffraction. Proc. Natl. Acad. Sci. USA.

[45] Vazquez-Fernandez E., Vos M.R., Afanasyev P., Cebey L., Sevillano A.M., Vidal E., Rosa I., Renault L., Ramos A., Peters P.J. (2016). The Structural Architecture of an Infectious Mammalian Prion Using Electron Cryomicroscopy. PLoS Pathog..

[46] Spagnolli G., Rigoli M., Orioli S., Sevillano A.M., Faccioli P., Wille H., Biasini E., Requena J.R. (2019). Full atomistic model of prion structure and conversion. PLoS Pathog..

[47] Baskakov I.V., Caughey B., Requena J.R., Sevillano A.M., Surewicz W.K., Wille H. (2019). The prion 2018 round tables (I): The structure of PrP^Sc^. Prion.

[48] Groveman B.R., Dolan M.A., Taubner L.M., Kraus A., Wickner R.B., Caughey B. (2014). Parallel in-register intermolecular beta-sheet architectures for prion-seeded prion protein (PrP) amyloids. J. Biol. Chem..

[49] Ferhadian D., Contrant M., Printz-Schweigert A., Smyth R.P., Paillart J.C., Marquet R. (2018). Structural and Functional Motifs in Influenza Virus RNAs. Front. Microbiol..

[50] Adler V., Zeiler B., Kryukov V., Kascsak R., Rubenstein R., Grossman A. (2003). Small, highly structured RNAs participate in the conversion of human recombinant PrP^Sen^ to PrP^Res^ in vitro. J. Mol. Biol..

[51] Deleault N.R., Lucassen R.W., Supattapone S. (2003). RNA molecules stimulate prion protein conversion. Nature.

[52] Ehresmann D.W., Hogan R.N. (1986). Acceleration of scrapie disease in mice by an adenovirus. Intervirology.

[53] Stanton J.B., Knowles D.P., O’Rourke K.I., Herrmann-Hoesing L.M., Mathison B.A., Baszler T.V. (2008). Small-ruminant lentivirus enhances PrP^Sc^ accumulation in cultured sheep microglial cells. J. Virol..

[54] Haviv Y., Avrahami D., Ovadia H., Ben-Hur T., Gabizon R., Sharon R. (2008). Induced neuroprotection independently from PrP^Sc^ accumulation in a mouse model for prion disease treated with simvastatin. Arch. Neurol..

[55] Leblanc P., Alais S., Porto-Carreiro I., Lehmann S., Grassi J., Raposo G., Darlix J.L. (2006). Retrovirus infection strongly enhances scrapie infectivity release in cell culture. EMBO J..

[56] Watts J.C., Prusiner S.B. (2017). Experimental Models of Inherited PrP Prion Diseases. Cold Spring Harb. Perspect. Med..

[57] Hsiao K.K., Scott M., Foster D., Groth D.F., DeArmond S.J., Prusiner S.B. (1990). Spontaneous neurodegeneration in transgenic mice with mutant prion protein. Science.

[58] Hsiao K.K., Groth D., Scott M., Yang S.L., Serban H., Rapp D., Foster D., Torchia M., Dearmond S.J., Prusiner S.B. (1994). Serial transmission in rodents of neurodegeneration from transgenic mice expressing mutant prion protein. Proc. Natl. Acad. Sci. USA.

[59] Yang W., Cook J., Rassbach B., Lemus A., DeArmond S.J., Mastrianni J.A. (2009). A New Transgenic Mouse Model of Gerstmann-Straussler-Scheinker Syndrome Caused by the A117V Mutation of PRNP. J. Neurosci..

[60] Chiesa R., Piccardo P., Ghetti B., Harris D.A. (1998). Neurological illness in transgenic mice expressing a prion protein with an insertional mutation. Neuron.

[61] Chiesa R., Drisaldi B., Quaglio E., Migheli A., Piccardo P., Ghetti B., Harris D.A. (2000). Accumulation of protease-resistant prion protein (PrP) and apoptosis of cerebellar granule cells in transgenic mice expressing a PrP insertional mutation. Proc. Natl. Acad. Sci. USA.

[62] Biasini E., Seegulam M.E., Patti B.N., Solforosi L., Medrano A.Z., Christensen H.M., Senatore A., Chiesa R., Williamson R.A., Harris D.A. (2008). Non-infectious aggregates of the prion protein react with several PrP^Sc^-directed antibodies. J. Neurochem..

[63] Friedman-Levi Y., Meiner Z., Canello T., Frid K., Kovacs G.G., Budka H., Avrahami D., Gabizon R. (2011). Fatal prion disease in a mouse model of genetic E200K Creutzfeldt-Jakob disease. PLoS Pathog..

[64] Asante E.A., Gowland I., Grimshaw A., Linehan J.M., Smidak M., Houghton R., Osiguwa O., Tomlinson A., Joiner S., Brandner S. (2009). Absence of spontaneous disease and comparative prion susceptibility of transgenic mice expressing mutant human prion proteins. J. Gen. Virol..

[65] Jackson W.S., Borkowski A.W., Watson N.E., King O.D., Faas H., Jasanoff A., Lindquist S. (2013). Profoundly different prion diseases in knock-in mice carrying single PrP codon substitutions associated with human diseases. Proc. Natl. Acad. Sci. USA.

[66] Dossena S., Imeri L., Mangieri M., Garofoli A., Ferrari L., Senatore A., Restelli E., Balducci C., Fiordaliso F., Salio M. (2008). Mutant prion protein expression causes motor and memory deficits and abnormal sleep patterns in a transgenic mouse model. Neuron.

[67] Bouybayoune I., Mantovani S., Del Gallo F., Bertani I., Restelli E., Comerio L., Tapella L., Baracchi F., Fernandez-Borges N., Mangieri M. (2015). Transgenic fatal familial insomnia mice indicate prion infectivity-independent mechanisms of pathogenesis and phenotypic expression of disease. PLoS Pathog..

[68] Jackson W.S., Borkowski A.W., Faas H., Steele A.D., King O.D., Watson N., Jasanoff A., Lindquist S. (2009). Spontaneous generation of prion infectivity in fatal familial insomnia knockin mice. Neuron.

[69] Fischer M., Rulicke T., Raeber A., Sailer A., Moser M., Oesch B., Brandner S., Aguzzi A., Weissmann C. (1996). Prion protein (PrP) with amino-proximal deletions restoring susceptibility of PrP knockout mice to scrapie. EMBO J..

[70] Turnbaugh J.A., Unterberger U., Saa P., Massignan T., Fluharty B.R., Bowman F.P., Miller M.B., Supattapone S., Biasini E., Harris D.A. (2012). The N-terminal, polybasic region of PrP^C^ dictates the efficiency of prion propagation by binding to PrP^Sc^. J. Neurosci..

[71] Das N.R., Miyata H., Hara H., Chida J., Uchiyama K., Masujin K., Watanabe H., Kondoh G., Sakaguchi S. (2020). The N-Terminal Polybasic Region of Prion Protein Is Crucial in Prion Pathogenesis Independently of the Octapeptide Repeat Region. Mol. Neurobiol..

[72] Flechsig E., Shmerling D., Hegyi I., Raeber A.J., Fischer M., Cozzio A., von Mering C., Aguzzi A., Weissmann C. (2000). Prion protein devoid of the octapeptide repeat region restores susceptibility to scrapie in PrP knockout mice. Neuron.

[73] Weissmann C., Flechsig E. (2003). PrP knock-out and PrP transgenic mice in prion research. Br. Med. Bull..

[74] Hara H., Miyata H., Das N.R., Chida J., Yoshimochi T., Uchiyama K., Watanabe H., Kondoh G., Yokoyama T., Sakaguchi S. (2018). Prion Protein Devoid of the Octapeptide Repeat Region Delays Bovine Spongiform Encephalopathy Pathogenesis in Mice. J. Virol..

[75] Uchiyama K., Miyata H., Yamaguchi Y., Imamura M., Okazaki M., Pasiana A.D., Chida J., Hara H., Atarashi R., Watanabe H. (2020). Strain-Dependent Prion Infection in Mice Expressing Prion Protein with Deletion of Central Residues 91–106. Int. J. Mol. Sci..

[76] Hara H., Okemoto-Nakamura Y., Shinkai-Ouchi F., Hanada K., Yamakawa Y., Hagiwara K. (2012). Mouse prion protein (PrP) segment 100 to 104 regulates conversion of PrP^C^ to PrP^Sc^ in prion-infected neuroblastoma cells. J. Virol..

[77] Hagiwara K., Hara H., Hanada K. (2013). Species-barrier phenomenon in prion transmissibility from a viewpoint of protein science. J. Biochem..

[78] Govaerts C., Wille H., Prusiner S.B., Cohen F.E. (2004). Evidence for assembly of prions with left-handed beta-helices into trimers. Proc. Natl. Acad. Sci. USA.

[79] Kraus A., Hoyt F., Schwartz C.L., Hansen B., Artikis E., Hughson A.G., Raymond G.J., Race B., Baron G.S., Caughey B. (2021). High-resolution structure and strain comparison of infectious mammalian prions. Mol. Cell.

